# Comparative proteomic analysis to annotate the structural and functional association of the hypothetical proteins of *S*. *maltophilia k279a* and predict potential T and B cell targets for vaccination

**DOI:** 10.1371/journal.pone.0252295

**Published:** 2021-05-27

**Authors:** Md. Muzahid Ahmed Ezaj, Md. Sajedul Haque, Shifath Bin Syed, Md. Shakil Ahmed Khan, Kazi Rejvee Ahmed, Mst. Tania Khatun, S. M. Abdul Nayeem, Golam Rosul Rizvi, Mohammad Al-Forkan, Laila Khaleda

**Affiliations:** 1 Department of Genetic Engineering and Biotechnology, Faculty of Biological Sciences, University of Chittagong, Chattogram, Bangladesh; 2 Reverse Vaccinology Research Division, Advanced Bioinformatics, Computational Biology and Data Science Laboratory, Chittagong, Bangladesh; 3 Department of Chemistry, Faculty of Science, University of Chittagong, Chattogram, Bangladesh; 4 Department of Biotechnology and Genetic Engineering, Faculty of Biological Sciences, Islamic University, Kushtia, Bangladesh; National Chiao Tung University College of Biological Science and Technology, TAIWAN

## Abstract

*Stenotrophomonas maltophilia* is a multidrug-resistant bacterium with no precise clinical treatment. This bacterium can be a vital cause for death and different organ failures in immune-compromised, immune-competent, and long-time hospitalized patients. Extensive quorum sensing capability has become a challenge to develop new drugs against this pathogen. Moreover, the organism possesses about 789 proteins which function, structure, and pathogenesis remain obscured. In this piece of work, we tried to enlighten the aforementioned sectors using highly reliable bioinformatics tools validated by the scientific community. At first, the whole proteome sequence of the organism was retrieved and stored. Then we separated the hypothetical proteins and searched for the conserved domain with a high confidence level and multi-server validation, which resulted in 24 such proteins. Furthermore, all of their physical and chemical characterizations were performed, such as theoretical isoelectric point, molecular weight, GRAVY value, and many more. Besides, the subcellular localization, protein-protein interactions, functional motifs, 3D structures, antigenicity, and virulence factors were also evaluated. As an extension of this work, ’RTFAMSSER’ and ’PAAPQPSAS’ were predicted as potential T and B cell epitopes, respectively. We hope our findings will help in better understating the pathogenesis and smoothen the way to the cure.

## Introduction

*Stenotrophomonas maltophilia* is a major emerging nosocomial pathogen [1] and is most commonly found in cystic fibrosis (CF) patients worldwide [2]. Among the multidrug-resistant organisms (MDROs), World Health Organization (WHO) enlisted *S*. *maltophilia* as one of the leading organisms found in the hospital settings [3] and causes nosocomial infection [4]. It is a Multi-Drug Resistant (MDR), gram-negative [5], ubiquitous [6], non-fermenting, bacilli [7] that form biofilms [8–11], which is responsible for 65% of infections that are acquired from hospitals [12]. *S*. *maltophilia* is generally found in plant roots, animals, and soils [13–19], dialysate sample and hemodialysis water [20], cannulae, nebulizer, dental units, prosthetic devices [21–26], ICU (Intensive Care Unit) [27] and airborne transmission can occur from the infected CF patients [28]. This pathogen causes a broad spectrum of infections including respiratory tract infections (RTIs), COPD (Chronic Obstructive Pulmonary Disease), pneumonia, biliary sepsis, bacteremia, bone and joint, soft tissues, and urinary tract infections, eye infections, endocarditis, endophthalmitis, meningitis [28–43]. Recent studies showed that it is the third most occurring (about 9.1%) NFGNB (Nonfermenting Gram-Negative Bacilli) [44] with an extremely high death rate of 14 to 69% in bacteremia patients [45]. The prevalence of the infections associated with this organism has increased from 0.8 to 1.68% during 1997–2012 [44]. It is a life-threatening pathogen to immunocompromised individuals, ICU patients, cancer patients, graft transferred patients [32, 46, 47], and immunocompetent persons as well [6]. The main problem to fight this organism is the multi-drug resistance acquired through DSF (Diffusible Signal Factor)-mediated quorum sensing [48] or horizontal gene transfer [15]. Trimethoprim-sulfamethoxazole (SXT) is widely used to fight this organism, which has less efficacy [49]. So, it is quite important to develop new drugs to eliminate this pathogen.

After the first isolation in 1943, *S*. *maltophilia* was named *Bacterium bookeri*, and further characterization renamed it to *Pseudomonas maltophilia* [50]. Cistron analysis of rRNA renamed it as *Xanthomonas maltophilia* [51], but later it was changed to *Stenotrophomonas maltophilia* in1993 based on the result of 16S rRNA genes [51, 52]. The complete genome of the well-characterized strain of *S*. *maltophilia K279a* was sequenced and analyzed in 2008 to improve our understanding of the biology of this low-grade pathogen [48]. The reference sequence of *S*. *maltophilia* 279A is stored in the NCBI (National Center for Biotechnology Information) database, which contains 4,851,126 bp long circular chromosome having 4490 genes encode 4332 proteins. The G+C content is 66.7, and it has 74 tRNAs [48].

When a protein is assumed to be encoded by a well-defined open reading frame (ORF), but no experimental protein product is identified or characterized, it is called Hypothetical Protein (HP) [53]. Most of the genomes contain about half of the HPs, which have proteomic and genomic significance [54, 55]. These HPs are believed to have crucial roles in the survival and progression of the diseases by the pathogens [53, 56]. New pathways, structures, functions cascades can be identified through precise annotation of these HPs [55], where novel ones can act as a marker or target for pharmaceutical uses [57, 58]. Among the proteins of *S*. *maltophilia*, about 789 proteins are of unknown functionalities and characters.

Several bioinformatics studies have been done on various microorganisms, i.e., *Candida dubliniensis* [56], *Haemophilus influenza* [59], *Clostridium tetani* [60], *Treponema pallidum ssp*. *Pallidum* [61] to analyze the HPs of these pathogens using the structure and sequence-based methods. But there is no evidence of such a study on *S*. *maltophilia*. As per our knowledge, this is the first study that provides a proper analysis of the functions and structures of conserved HPs of *S*. *maltophilia*.

Here we will be using different bioinformatics tools to predict the functions, structures, physicochemical properties, subcellular localizations, antigenicity, virulence factors, and some other phenomena of the HPs of *S*. *maltophilia*. Furthermore, we will also predict the best epitope-based subunit vaccine candidate and different B and T cell epitopes.

## Materials and methods

**Fig 1:**
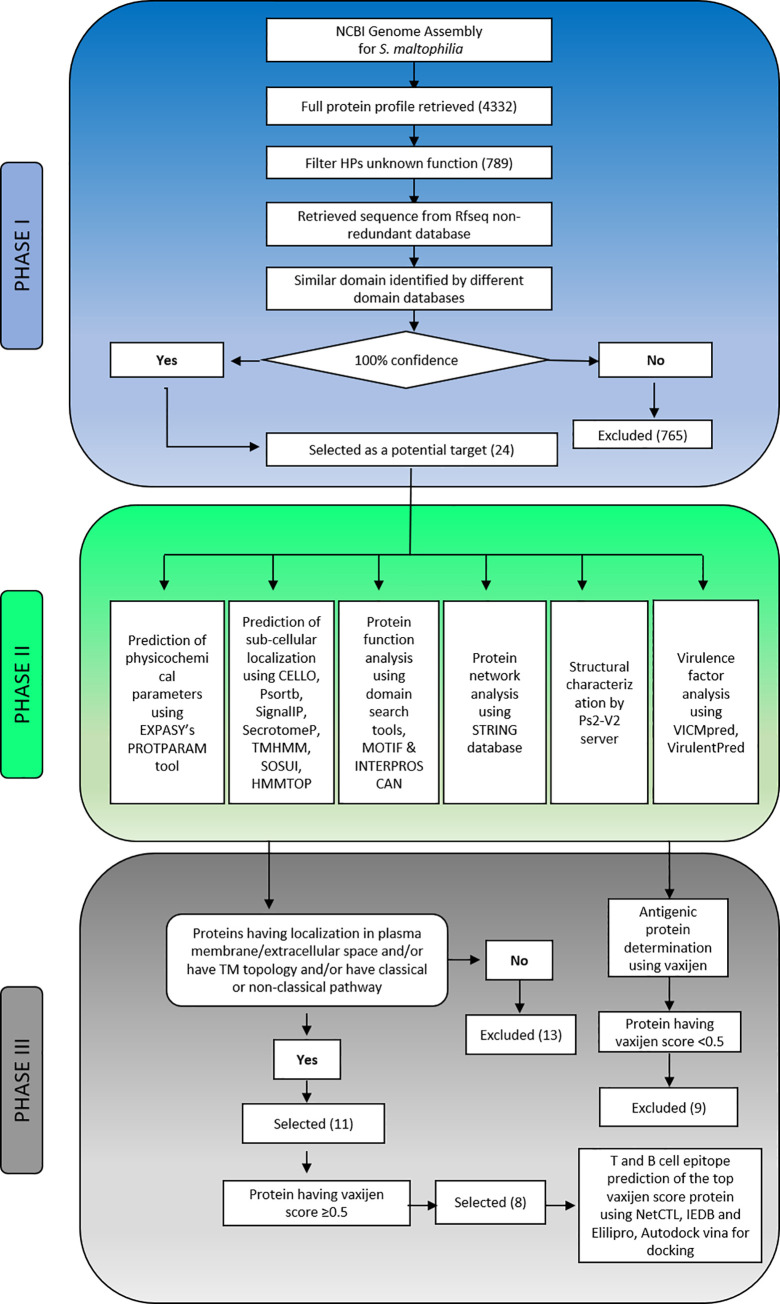
The complete framework of the study was used to annotate the functions of the 24 HPs from *S. maltophilia*.

The complete framework and the tools used in this study are depicted in **Fig 1** and **Table 1**, respectively. The whole process is comprised of three phases: Phase-I, Phase-II, and Phase-III. The genome analysis and characterization of the HPs are performed in Phase-I. Phase-II includes annotations of different functional properties using multiple servers and tools. Prioritization of targets to design a vaccine against the pathogen and validation of the findings are illustrated in Phase-III.

**Table 1:** List of the bioinformatics databases and tools used in this study.

Objective	Serial No.	Tools	URL	Remarks
**Physicochemical Characterization**	1.	ProtoParam	http://web.expasy.org/protparam/	This server predicts different physical and chemical properties of accuracy sequence
**Subcellular Localization**	1.	CELLO	http://cello.life.nctu.edu.tw	Prediction by this server is 91% accurate
	2.	PSORT B	http://www.psort.org/psortb	The result is 97% precise
	3.	SignalP	http://www.cbs.dtu.dk/services/SignalP/	SignalP predicts the cleavage site of signal peptide
	4.	SecretomeP	http://www.cbs.dtu.dk/services/SecretomeP/	This server is used to predict non-classical secretion
	5.	HMMTOP	http://www.enzim.hu/hmmtop/	Used for transmembrane topology prediction
	6.	TMHMM	http://www.cbs.dtu.dk/services/TMHMM/	Predicts membrane topology
	7.	SOSUI	http://bp.nuap.nagoya-u.ac.jp/sosui/sosui_submit.htm	Predicts whether a protein is transmembrane or soluble
**Conserved Domain and Function Prediction**	1.	CDD-BLAST	http://www.ncbi.nlm.nih.gov/Structure/cdd/wrpsb.cgi	The conserved domain search tool in the query sequence
	2.	SMART	http://smart.embl-heidelberg.de/	Predicts domains in the protein sequence
	3.	PFAM	http://pfam.xfam.org/search	Uses multiple sequence alignment to search protein family
	4.	ScanProsite	http://prosite.expasy.org/scanprosite/	Scans protein based on the motif, domain, and pattern
	5.	(PS)2-v2	http://ps2.life.nctu.edu.tw/	Predicts 3D structure
**Motif Discovery**	1.	MOTIF	http://www.genome.jp/tools/motif/	Motif discovery tool of Japanese GenomeNet service
	2.	INTERPROSCAN	http://www.ebi.ac.uk/InterProScan/	Motif is searched in the InterPro
**Virulence Prediction**	1.	VirulentPred	http://bioinfo.icgeb.res.in/virulent/	Accuracy is 81.8%
	2.	VICMpred	http://www.imtech.res.in/raghava/vicmpred/	Predicts virulence factor with 70.75% accuracy
**Protein-Protein Interaction**	1.	STRING	http://string-db.org	keeps the data of different protein-protein interaction network
**T Cell Epitope Prediction**	1.	NetCTL 1.2	http://www.cbs.dtu.dk/services/NetCTL/	Predicts potential T cell epitopes
	2.	IEDB T cell epitope prediction tools	http://tools.iedb.org/main/tcell/	Prediction of T cell epitopes with high accuracy
	3.	Population coverage	http://tools.iedb.org/population/	Predicts the population coverage of the epitopes
**B Cell Epitope Prediction**	1.	Antibody Epitope Prediction	http://tools.iedb.org/bcell/	This server predicts linear B cell epitopes using protein sequence
	2.	ElliPro	http://tools.iedb.org/ellipro/	Conformational B cell epitopes are predicted using a PDB file
**Allergenicity Assessment**	1.	AllerTOP 2.0	https://www.ddg-pharmfac.net/AllerTOP/	Predicts allergenicity
	2.	AllerCatPro	https://allercatpro.bii.a-star.edu.sg/	Overall accuracy is 84%

### Phase-I

#### Sequence retrieval

The complete genome sequence of *S*. *maltophilia K279a* (GeneBank Assembly ID: GCF_000072485.1) (RefSeq: NC_010943.1) was retrieved from the NCBI database (http://www.ncbi.nlm.nih.gov/). There were about 789 Hypothetical Proteins (HPs) among the 4332 proteins, which were separated and stored as FASTA files using the Refseq accession number for further analysis. Different computational strategies were followed to predict various essential properties of those proteins.

#### Conserved domain search

The repetitive sequences or recurring structural units that have notable functional capabilities in many contexts of a protein and thought to modulate or signify different functions in different proteins through their unique re-combinational rearrangements can be called domains [62]. Throughout evolution, these domains work as building blocks that are rigidly conserved among the protein families rather than the whole protein sequences [63].

To classify the protein families and to predict the highly conserved and well-defined domains or folds in the HPs, we used four online bioinformatics tools, namely CDD-BLAST [64–66], SMART [67], PFAM [68], ScanProsite [69]. All the HPs were subjected to those web tools mentioned above, which resulted in variable predictions of the conserved domains among the HPs. Thereat, variability was observed in the confidence level of the cumulative predictions. Percentile confidence scores were given to determine a higher or lower confidence level, i.e., a combinatorial score of 100 is given to those proteins which are being predicted to have the same functional domains. After analyzing all the HPs, we have found 24 proteins that have a high confidence level (HCL) of 100 and considered them for further investigations.

To find out highly conserved domains of a query protein, NCBI’s online tool CDD-BLAST uses RPBLAST, which is derived from PSI-BLAST, scans the query sequence with the help of Position Specific Scoring Matrices (PSSMs) against the Conserved Domain Database. The SMART stands for Simple Modular Architecture Research Tool, which is a web-based server that predicts domain profiles and architectural similarities of the target protein using stable Ensembl [70], SP-TrEMBL [71], Swiss Prot [72], where direct similarities search among the sequences is avoided. Pfam database has two parts: Pfam-A and the Pfam-B. The Pfam-A is derived from Pfamseq, which is built from the updated release of UniProtKB at a particular time frame. Each family of Pfam-A contains three major elements, namely: A curated seed alignment, a full alignment that is automatically constructed, and Profile Hidden Markov Models (Profile HMMs). On the contrary, the Automatic Domain Decomposition Algorithm (ADDA) generates low-quality un-annotated Pfam-B families using nonredundant clusters [73]. Most of the proteins of a set of an enormous number of proteins that are functionally different can be grouped into a narrow range of families according to their sequence similarities. This principle is the core of the web-based prediction tool ScanProsite.

### Phase-II

#### Physicochemical characterization

Different physicochemical properties of the HCL proteins were measured using Expasy’s ProtParam [74] server, i.e., theoretical isoelectric point (pI), molecular weight (MW), total amino acid number, Aliphatic index (AI) [75], extinction coefficient [76], grand average of hydropathy (GRAVY) [77], the total number of positive and negative charged residues and instability index [78].

#### Subcellular localization

Depending upon different positional orientations, a protein can be targeted for vaccines (structural or extracellular proteins) or drugs (cytoplasmic or intracellular proteins) [79], where UniProtKB can be useful for experimental proteins information [80]. There is an information gap about the HPs as they are not experimentally characterized. Hence, their subcellular localizations are also in concealment. To unveil this characteristic feature, online bioinformatics tools CELLO (v2.5) [81], which uses a system based on two-level SVM (Support Vector Machine) and PSORTb [82], which is the most reliable subcellular localization prediction tool for bacteria, was used. Besides, to predict signal peptide and secretory pathway (non-classical), we used the neural network-based system SignalP [83] and CBS server’s tool SecretomeP [84], respectively. Our study also used SOSUI [85], TMHMM [86], and HMMTOP [87] to predict the solubility and transmembrane topology of the proteins.

#### Domain and function assignment

To predict the precise functions of the proteins, we employed several servers for the accuracy of the work. CDD (Conserved Domain Database), ScanProsite, SMART, and Pfam were used earlier to search the domains. Furthermore, to assign functional motifs, the online tool MTIF (https://www.genome.jp/tools/motif/) was recruited where the output is very large. We also used InterProScan [88], which works in a combination of different signature recognition methods of proteins, utilizing InterPro consortium, where large databases like Pfam, SUPERFAMILY, SMART, PANTHER, ProSite are the integral parts.

#### Protein structure prediction

Along with the functional motif prediction of the HPs, it is crucial to predict the 3D structures as well [89]. Template-based protein structure prediction online tool PS square version 2, popularly known as PS2-v2 [90], was exploited to predict the tertiary structure of the proteins. Protein FASTA sequence is the input format for the query, which is analyzed using both Pair-wise and multiple sequence alignments in the combination of IMPALA [91], PSI-BLAST [64, 92], T-Coffee [93] through both target-template selection and alignment. By default, the best homologous template is selected based on scores to generate a 3D structure using the amino acid sequence of the target protein with the help of an integrated modeling package. However, the server failed to generate a 3D model for some proteins. To overcome this problem, we implemented the manual system to select a template from the suggested list of the server and generated the 3D models of those proteins. Though it was executed successfully still, there was an error in template selection and modeling for two proteins. We overcame this problem using the SWISS-MODEL [94]. All the predicted results for the HCL HPs were stored in PDB (Protein Data Bank) format.

#### Virulence factor analysis

Virulence Factors (VFs) are related to the intensity or severity of an infection and are targeted for drug development. More the virulence, more the potency as the target for drugs [95]. To determine the VFs of HCL HPs, VirulentPred [96] with an accuracy of 81.8% and VICMpred [97] with the corresponding accuracy of 70.75% were used in this study. Both servers use a fivefold cross-validation strategy with the SVM method.

#### Functional protein association and PPI prediction

STRING [98] uses four sources: Previous Knowledge, (Conserved) Co-expression, High-throughput Experiment, and Genomic Context to predict Protein-Protein Interactions (PPI). We completed the prediction with STRING v11 [99], where only the highest scored protein was taken as a functionally associated partner. Besides, this study also showed the PPI network and gene co-occurrence for the highest antigenic and all the virulent proteins.

#### Identification of antigenic protein

All the previous analyses helped us to select 11 proteins among the entire set of HCL HPs, which are predicted to be connected to classical or non-classical secretory pathways or localized in the extracellular space/periplasm/plasma membrane by CELLO prediction server or possessed one or more transmembrane topology. These types of proteins are generally targeted for subunit vaccines. To check the probability of these proteins as potential protective antigens, we used the VaxiJen v2.0 [100] server at a threshold of 0.5 for a very high precisions level. Besides, we also checked the antigenicity of the rest of the proteins as they can also induce cell-mediated and humoral immunity [101], and we found about 15 proteins to be antigenic out of the 24. Among them, the most antigenic protein was taken to predict potential B cell and T cell epitopes.

### Phase-III

#### T cell epitope identification

To induce cell-mediated and humoral immunity, identifying potential epitopes for T cell and B cell is essential. A tool from the CBS server, NetCTL 1.2 [102], was used at threshold 0.5 with a sensitivity of 0.89 and specificity of 0.94 to predict probable epitopes. The prediction is based on the peptide to MHC-I (Major Histocompatibility Complex class I) binding, C terminal proteasomal cleavage, and TAP (Transporter associated with Antigen Processing) transport efficiency using 12 prominent supertypes of MHC-I. This server uses ANN (Artificial Neural Network) based method to predict MHC-I binding and C terminal proteasomal cleavage where TAP transport efficiency is calculated using the Weight Matrix method.

For peptide to MHC-I binding prediction, the Stabilize Matrix Method (SMM) [103] was selected in a tool from IEDB (Immune Epitope Database) [104], which was employed to determine the IC_50_ (Half Maximal Inhibitory Concentration) value. All the available alleles were selected with the peptide length of 9.0 before the prediction. Finally, selected epitopes were analyzed using the T cell epitopes-processing prediction tool that calculates a combinatorial score for TAP transport, proteasomal cleavage, and MHC-I binding [105]. We used the SMM method in this case as well.

#### Prediction of population coverage

Among the different ethnicity, the coverage of our proposed epitopes with corresponding HLAs was calculated using the population coverage tool [106] from the IEDB server.

#### Allergenicity appraisal

Two web-based tools were used to predict the allergenicity of the epitopes with very high specificity, namely: AllerTOP v2.0 [107] with an accuracy of 85.3% and AllerCatPro [108] with 84% accuracy.

#### Molecular docking simulations

Before docking, the 3D structure of the epitope RTFAMSSER was built using PEP-FOLD3 [109], and the PDB (Protein Data Bank) structure of the HLA-C*03:03 (PDB ID: 1EFX) was retrieved from the RCSB database [110] where it was complexed with human natural killer cell receptor KIR2DL2. Then the complex was opened using Discovery Studio [111] to remove the receptor and recover the simplified HLA-C*03:03.

Autodock Vina [112] was used to calculate the binding energy between the target epitope and the corresponding HLA. The docked complex was visualized using PyMol [113] and UCSF Chimera [114].

However, the rest of the epitopes and HLA alleles were also subjected to molecular docking simulation following the similar procedure in order to estimate the relation between the docking score, IC50 value, and combined score of proteasome score, TAP score, MHC-I score, processing score.

#### Linear and conformational b cell epitope identification

B lymphocytes play a crucial role in the induction of immune response mediated by B cell epitopes [115]. We used IEDB B cell epitope prediction tools to identify the B cell epitopes. Bepipred linear epitope prediction analysis [116], Kolaskar and Tongaonkar antigenicity scale [117], Karplus and Schulz flexibility prediction [118], Emini surface accessibility prediction [119], Parker hydrophilicity prediction [120] were performed to predict and confirm the linear antigenic B cell epitope properties. As beta-turn regions of a protein are found in the antigenic portions [121], we utilized the Chou and Fasman beta-turn prediction tool in this regard [122].

Furthermore, the conformational or discontinuous B cell epitopes were also predicted using the IEDB tool Elipro [123]. For this prediction, the 3D structure of the protein was built using PS2-v2 and validated. Then the valid, optimized structure was submitted to the server, and the scoring criteria were set at 0.5, where less than that value is rejected, and the most stringent score is considered to be at 1.0. To calculate the residue clusters, 6.0 Å (Angstrom) was selected as the maximum distance parameter.

## Result and discussion

### Sequence evaluation

The implementation of advanced technologies in DNA sequencing techniques enables us to reveal the exact sequence of an immense number of bacterial genomes in a short time with a considerably low cost. Many genes are found to be conserved in a broad spectrum of bacterial genomes throughout the evolutionary process. As a result, the precise annotations and functions of these genes are assigned using sequence homology or similarity search against functionally specified genes [124]. Although, one-third of the sequenced genes have no specified functional assignment due to the rapid deviations of functions between the similar gene sequences in the road to evolution [125, 126]. Consequently, only sequence homology or similarity search cannot predict or ascertain the proper function of a gene, which ultimately results in faulty functional allocation [127].

To overcome this crux and lessen the proportion of HPs, it is recommended to use multiple bioinformatics tools for discovering appropriate functions of the hypothetical proteins. On account of this, the current study focused on annotating the functions of the hypothetical proteins of *Stenotrophomonas maltophilia* by recruiting diverse bioinformatics methods and tools. At first, the conserved domains for the 789 hypothetical proteins were searched with the help of four bioinformatics web tools, namely Pfam, CDD-BLAST, ScanProsite, SMART. Based on these results, the proteins were classified into five groups where 24 proteins showed a specific consensus functional domain in all the tools and hence are grouped into high confidence level (HCL) proteins. The tools did not find any domain for 479 proteins, and the combined confidence level was zero. Remaining HPs (286 proteins) showed hit in one, two, or three of the four tools mentioned above, which resulted in different confidence levels (i.e., 25% for 172, 50% for 59, and 75% for 55 proteins). The result is summarized in the **S1 Table**. However, further analysis is required to reveal the proper functions of these proteins. We considered only the 24 HCL HPs for downstream study because these proteins showed at least one conserved domain in all four servers. To avoid false-positive results and increase the accuracy of the study, we excluded all the other four confidence level proteins.

The theoretical pI, molecular weight, extinction coefficient, total number of negative and positive charged residues, instability index, GRAVY value, and other physiochemical properties of the HCL HPs were measured by the online bioinformatics tool ProtParam, and the result is shown in **S2 Table**. The cumulative value of hydropathy for all the amino acid residues of a protein chain is divided by the total number of residues of that protein sequence to calculate the GRAVY value [77]. The lower GRAVY value indicates the possibility of a protein being hydrophilic (globular), where the higher value confirms the hydrophobic (membranous) nature of the proteins.

We found the GRAVY values of our concerned proteins ranging from -0.958 to -0.044, which points towards the hydrophilic properties of the proteins and helps in predicting the localization. Functional motifs of these hypothetical proteins were discovered using web-based tools MOTIF and InterProScan for further confirmation about the functions. Using the tertiary structural information, we can validate the predicted biochemical functions of a protein [128]. So, we assigned the PS2-v2 server for the resolution of the 3D structure of HCL HPs, which generates a PDB file in a template-based manner and fold recognition scheme. Then all the sequence evaluation data were collated, and the HCL HPs were sorted into different functional groups, which consist of eleven enzymes, three binding proteins, four regulatory proteins, two inhibitors, two transporters, and two proteins of manifold functions. These groups are described below:

#### Enzymes

Bacterial enzymes are crucial for their pathogenesis in the host. They also provide essential nutrients and control various metabolic pathways, which helps in the growth and survival of the organism [129]. In our study, we found 11 enzymes among the 24 annotated HCL HPs that have different physiological and pathological importance to *S*. *maltophilia*. Among them, WP_005408386.1 and WP_012479842.1 are phosphotransferases (catalyze phosphorylation reactions), which play a key role in the bacterial PTS (Phosphotransferase System) in transporting sugar [130]. Besides, WP_012479842.1 is a member of the chloramphenicol phosphotransferase-like protein family. This protein phosphorylates and inactivates the lethal chloramphenicol metabolites in bacteria, which inhibits ribosomal peptidyl transferase and thus shuts protein production down [131, 132].

We found WP_005409007.1 protein to be a member of the SmrA superfamily. Member of this family contains the Smr domain, which is thought to participate in crossing over, mismatch repair, or segregation, and it also has nicking endonuclease activity [133, 134]. Vicinal Oxygen Chelate (VOC) is a family of proteins that are involved in sequestering and localizing metal ions. This type of domain or fold consists of two β-α-β-β-β units, which are responsible for the formation of the partially closed beta-sheet barrel around the metal ions [135]. The protein WP_005414366.1 was found to be a member of the VOC superfamily. So, we assume this protein may involve in the metal resistance trait in the organism. The protein WP_012478637.1 contains the Haloacid Dehydrogenase or HAD domain superfamily, which participates in various cellular processes, i.e., detoxification, amino acid biosynthesis, and many more [136, 137]. X-ray crystallography revealed the conserve hydrolase fold analogous to the Rossmann fold found in the members of this superfamily [138]. This fold contains two subdomains, where the large one remains strictly conserved, and the small domain shows structural variations among the classes [139]. WP_012480920.1 protein belongs to the Isoprenoid Biosynthesis Enzymes Class-I.

Protein WP_012478648.1 was found to maintain the protein tyrosine phosphatase superfamily, which is homologous to the dual-specificity protein phosphatase known as Cyclin-Dependent Kinase Inhibitor-3 (CDKN3) [140]. WP_012480806.1 glycosidase enzyme possesses six helical hairpin structures in a closed circular order and hence are included in the six-hairpin glycosidase superfamily [141]. We found the CheB domain in WP_012481043.1 protein, which is a strong indication for this protein of being a member of methylesterase CheB, C-terminal superfamily. The members of this superfamily consist of parallel β sheet with the α-β-α array in seven strands and remove the methyl group from the methyl-accepting chemotaxis proteins (MCP) [142, 143]. Among the enzymes, we were able to identify only one protease enzyme (WP_044570756.1) containing DUF2268 (DUF is annotated as Domain of Unknown Function) domain, which is predicted as a Zn-dependent protease.

#### Binding proteins

We have characterized two (WP_005412620.1 and WP_005413412.1) calcium ion binding proteins containing the EF-hand domain, and the rest is a DNA binding protein. EF-hand Ca^2+^-binding motifs are found in pairs. Each of them comprises a loop that is 12 residues long where a 12 residue α-helix flanks the loop on either side [144]. The conformation of the EF-hand motif changes upon the binding of the Ca^2+^ ion. The ion is positioned in the loop in a pentagonal bipyramidal fashion [145, 146]. The DNA binding protein WP_012479848.1 belongs to the Bro-N family proteins which function is unknown. But the experimental shreds of evidence of Bro-A and Bro-C suggest its ability to regulate host DNA replication and/or transcription by binding with it directly [147].

#### Regulatory proteins

In this study, we were successfully able to characterize a novel regulatory protein (WP_012479796.1) of *S*. *maltophilia* that is crucial for its extensive multi-drug resistance nature. This protein is a member of the LuxR transcription regulatory protein family, which is one of the most important proteins in Quorum Sensing (QS). It also plays key roles in plasmid transfer, motility, biofilm formation, nodulation, and the expression of many genes that includes the antibiotics and virulence factors encoding genes [148]. This family protein has an autoinducer binding domain at the N-terminal that generally binds to the N-acyl homoserine lactones (AHL). Binding with autoinducer results in the dismantling of the C-terminal DNA-binding domain that promotes it to bind with the DNA and actuate the transcription [149].

WP_012479125.1 and WP_012480949.1 protein contains the structural motif Tetratrico Peptide Repeat (TPR). This protein domain consists of 34 amino acids that are repeated 3–16 fold and occur in a helix-turn-helix array with the nearby TPRs in a parallel manner, which results in anti-parallel α-helices [150, 151]. These proteins are engaged in many biological processes, such as the regulation of transcription, cell cycle, protein transport, and folding [152].

The functional analysis disclosed a vital protein (WP_012478875.1) that can act as a regulatory protein and immune protein both at the same time due to the presence of Ankyrin repeat-containing domain and NTF2 fold domain. NTF2 domain-containing proteins are found in the polymorphic toxin system of bacteria [153]. This domain is always fused with ankyrin repeats, which is a multi-repeat β_2_-α_2_ motif of 33 amino acid residues [154]. Proteins of these domains can participate in a variety of functions, including the initiation of transcription, ion transportation, cell-cycle regulation, and signal transduction [155].

#### Inhibitor proteins

Two HCL HPs among the annotated 24 showed similarities with lysozyme inhibitors. Lysozymes are the hydrolase enzymes recruited by the innate immune system of animals for the degradation of bacterial major cell wall component peptidoglycan [156]. WP_005413200.1 is a C-type lysozyme inhibitor superfamily protein, more specifically membrane-bound lysozyme inhibitor of C-type lysozyme (MliC), which are well known for their conferring support in extensive lysozyme tolerance to the gram-negative bacteria [157]. This protein forms ionic and hydrogen bonds with its invariant loop to the lysozyme at the active site cleft [158]. The second inhibitor (WP_044569343.1) is of the IVY (Inhibitor of Vertebrate Lysozyme) superfamily, which is also known as a virulence factor [159, 160]. IVY proteins consist of three layers of α_2_-β_5_-α_2_ topology and a crucial 5-residue long loop for the inhibitory function [161].

#### Transporter proteins

Maintenance and assembly of outer membrane (OM) components play a vital role in bacterial survival and pathogenesis. To aid this process, many transport proteins are involved in bacteria. We found two such proteins, namely the LPS-assembly lipoprotein LptE (WP_005410539.1) and Curli production assembly/transport component CsgG (WP_032966398.1). During the assembly through the beta-barrel assembly machine, LptE interacts with LptD and forms a complex [162] that is involved in lipopolysaccharides (LPS) assembly at the outer region of OM [163–165]. Along with them, LptA, LptB, and LptC are also involved in the LPS transport machinery. Blocking any of them disrupts the LPS assembly system as a whole and creates the same type of OM biogenesis defects [164]. On the other hand, CsgG is a lipoprotein that works as the stabilizer for CsgA and CsgB during the Curli assembly [166]. The Curli protein is amyloid fiber in nature and promotes cell to cell communication via biofilm formation [167].

We found two proteins showing miscellaneous functions. One of them (WP_024956629.1) contains the DUF2329 domain, which is a domain of unknown functionality. But WP_005410716.1 proteins were found to have a CheW like domain associated with the chemotaxis process of the bacteria [168]. The domain is about 150 residues long and is made up of two β-sheet subdomains. Every beta-sheet is comprised of a five-stranded loose beta-barrel centering a hydrophobic core component [169].

The MOTIF and InterProScan servers were used to validate the predicted functions of the proteins by the blast servers (**Table 2**). Web-based tool STRING was employed to predict the possible functional partners of the HCL HPs (**S3 Table**).

**Table 2:** Functional domains present in the HCL HPs.

Serial No.	Protein Accession No.	UniProt Id	MOTIF	INTERPROSCAN
**1**	WP 005408386.1	J7V4Q1	PTS system fructose IIA component	PTS EIIA man-typ sf, PTS EIIA man-typ
**2**	WP 005409007.1	J7VKL1	Smr Domain	Smr dom sf, Smr dom
**3**	WP 005410539.1	B2FPR6	Lipopolysaccharide-assembly, Prokaryotic membrane lipoprotein lipid attachment site	LPS assembly LptE
**4**	WP 005410716.1	J7VVQ8	CheW-like domain	CheW-like dom sf, CheW-like dom, CheW
**5**	WP 005411349.1	B2FKP0	Variant SH3 domain, SH3 domain, Bacterial SH3 domain, Protein of unknown function (DUF2442)	UCP034961 SH3 2, SH3-like dom sf, SH3 domain
**6**	WP 005412620.1	B2FTC2	EF-hand, Secreted protein acidic and rich in cysteine Ca binding region, Dockerin type I domain	EF Hand 1 Ca BS, EF-hand dom, EF-hand-dom pair
**7**	WP 005413200.1	B2FQ57	Membrane-bound lysozyme-inhibitor of c-type lysozyme	MliC sf, MliC
**8**	WP 005413412.1	B2FS21	EF-hand, Secreted protein acidic and rich in cysteine Ca binding region, Bacillus PapR protein, Peptidase propeptide, and YPEB domain	EF-hand-dom pair, EF-hand dom, EF Hand 1 Ca BS
**9**	WP 005414366.1	T5KJF3	Glyoxalase/Bleomycin resistance protein/Dioxygenase superfamily, Glyoxalase-like domain, YtxH-like protein	VOC, Glyas Bleomycin-R OHBP Dase, Glyas Fos-R dOase dom
**10**	WP 012478637.1	B2FT99	NLI interacting factor-like phosphatase	HAD-like sf, FCP1 dom, HAD sf
**11**	WP 012478648.1	B2FU04	Cyclin-dependent kinase inhibitor 3 (CDKN3), Protein-tyrosine phosphatase, Dual specificity phosphatase, catalytic domain, Tyrosine phosphatase family	CDKN3, Prot-tyrosine phosphatase-like, TYR PHOSPHATASE dom, Tyr Pase cat, PTPase domain
**12**	WP 012478875.1	B2FJ12	Ankyrin repeat, NTF2 fold immunity protein	Ankyrin rpt, Ankyrin rpt-contain sf, Imm-NTF2, Ankyrin rpt-contain dom
**13**	WP 012479125.1	B2FNJ7	Bacteriophage N adsorption protein A C-term, TPR repeat, Tetratricopeptide repeat, Alkyl sulfatase dimerization	TPR-contain dom, TPR-like helical dom sf, TPR repeat, NfrA C
**14**	WP 012479796.1	B2FLZ2	Bacterial regulatory proteins; luxR family, Autoinducer binding domain, Sigma-70; region 4, Helix-turn-helix domain, Homeodomain-like domain, HTH DNA binding domain, ECF sigma factor, PucR C-terminal helix-turn-helix domain, LexA DNA binding domain, Winged helix-turn-helix DNA-binding	Tscrpt reg LuxR HchA-assoc, TF LuxR autoind-bd dom sf, WH-like DNA-bd sf, Sig transdc resp-reg C-effector, Tscrpt reg LuxR C
**15**	WP 012479842.1	B2FM42	D5 N terminal like, Chloramphenicol phosphotransferase-like protein	Phage/plasmid primase P4 C, TOPRIM DnaG/twinkle, Helicase SF3 DNA-vir, DNA primase phage/plasmid
**16**	WP 012479848.1	B2FM48	BRO family, N-terminal domain, Phage antirepressor protein KilAC domain, Protein of unknown function DUF99	BRO N domain
**17**	WP 012480806.1	B2FM94	F5/8 type C domain, Amylo-alpha-1,6-glucosidase	FA58C, Galactose-bd-like sf, 6hp glycosidase-like sf, 6-hairpin glycosidase sf
**18**	WP 012480920.1	B2FP37	Polyprenyl synthetase	Isoprenoid synthase dom sf, Polyprenyl synt, Polyprenyl synt CS
**19**	WP 012480949.1	B2FPT9	Tetratricopeptide repeat, Transglutaminase-like superfamily, TPR repeat, MIT (microtubule interacting and transport) domain, BRO1-like domain, Anaphase-promoting complex, cyclosome, subunit 3	TPR-like helical dom sf, Papain-like cys pep sf, TPR repeat, Transglutaminase-like, TPR-contain dom
**20**	WP 012481043.1	B2FR32	CheB methylesterase	CheB C, Sig transdc resp-reg Me-estase
**21**	WP 024956629.1	A0A0U5DG84	Putative glucoamylase	DUF2329, UCP028431
**22**	WP 032966398.1	UPI0002E8A010	Curli production assembly/transport component CsgG, Peptidoglycan-synthase activator LpoB, Flagellar assembly protein T; middle domain	Curli assmbl/transp-comp CsgG
**23**	WP 044569343.1	UPI00031F6529	Inhibitor of vertebrate lysozyme (Ivy)	Inhibitor vert lysozyme sf
**24**	WP 044570756.1	UPI00031EA029	Predicted Zn-dependent protease (DUF2268)	DUF2268

### Structure prediction

All of the 24 HCL HPs were subjected to the PS2-v2 server to generate the 3D structure of the proteins. The server effectively produced a PDB file for each of the 22 proteins. In the case of the rest two proteins, it showed an error message, which is due to the inappropriate or unavailability of a suitable template for the prediction. To solve this problem, we used the SWISS-MODEL and generated the 3D structure. The result is depicted in the **S3 Table**.

### Subcellular localization prediction

The subcellular localization of the HCL HPs was predicted using various bioinformatics tools, which predicted not only their cellular locus but also their solubility and secretion or signaling ability along with possible membrane helices. Among the 24 HCL HPs we found about 10 proteins (WP_005412620.1, WP_005413200.1, WP_005413412.1, WP_012479125.1, WP_012480806.1, WP_012480949.1, WP_024956629.1, WP_032966398.1, WP_044569343.1, WP_044570756.1) that are in or near the outer membrane or the periplasmic space of *S*. *maltophilia*. All of them have at least one transmembrane helix to anchor the membrane. The remaining 14 proteins were predicted as cytoplasmic soluble proteins with no transmembrane helices. An exception of them is the protein WP_005410539.1. This protein possesses one transmembrane helix, which was further verified by all three tools (HMMTOP, TMHMM, and SOSUI). The result of subcellular localization is shown in the **S4 Table.**

### Virulent protein prediction

Virulentpred and VICMpred were used to predict the virulence factor of the high confidence level hypothetical proteins. These web tools predicted two HPs among the 24 proteins as virulent, and the other proteins were either non-virulent or predicted virulent by only one server. The result is shown in **Table 3**. It is thought that the virulence factors can be potentially good candidates and can provide comparatively better therapeutic interposition in case of bacterial infections [170]. Characterized virulent HPs can yield a dynamic target-based therapy against the infections and can be a subsidiary therapy to the antibiotics or can work as effector molecules to the immune response of the host [171].

**Table 3:** The virulence factor prediction result of the HPs of *S*. *maltophilia*.

Serial No.	Accession No	UniProt ID	VICMpred	Virulentpred
**1**	WP_005408386.1	J7V4Q1	Metabolism Molecule	Virulent
**2**	WP_005409007.1	J7VKL1	Metabolism Molecule	Non-Virulent
**3**	WP_005410539.1	B2FPR6	Metabolism Molecule	Virulent
**4**	WP_005410716.1	J7VVQ8	Cellular process	Virulent
**5**	WP_005411349.1	B2FKP0	Metabolism Molecule	Virulent
**6**	WP_005412620.1	B2FTC2	Metabolism Molecule	Virulent
**7**	WP_005413200.1	B2FQ57	Cellular process	Non-Virulent
**8**	WP_005413412.1	B2FS21	Metabolism Molecule	Virulent
**9**	WP_005414366.1	T5KJF3	Cellular process	Non-Virulent
**10**	WP_012478637.1	B2FT99	Metabolism Molecule	Non-Virulent
**11**	WP_012478648.1	B2FU04	Metabolism Molecule	Non-Virulent
**12**	WP_012478875.1	B2FJ12	Cellular process	Non-Virulent
**13**	WP_012479125.1	B2FNJ7	Information and storage	Non-Virulent
**14**	WP_012479796.1	B2FLZ2	Virulence factors	Virulent
**15**	WP_012479842.1	B2FM42	Cellular process	Non-Virulent
**16**	WP_012479848.1	B2FM48	Cellular process	Non-Virulent
**17**	WP_012480806.1	B2FM94	Virulence factors	Non-Virulent
**18**	WP_012480920.1	B2FP37	Metabolism Molecule	Virulent
**19**	WP_012480949.1	B2FPT9	Virulence factors	Virulent
**20**	WP_012481043.1	B2FR32	Cellular process	Non-Virulent
**21**	WP_024956629.1	A0A0U5DG84	Metabolism Molecule	Non-Virulent
**22**	WP_032966398.1	UPI0002E8A010	Cellular process	Non-Virulent
**23**	WP_044569343.1	UPI00031F6529	Metabolism Molecule	Non-Virulent
**24**	WP_044570756.1	UPI00031EA029	Metabolism Molecule	Non-Virulent

### Antigenic protein prediction

Antigenicity of a protein is the primary requirement of being targeted by the host immune system [172]. Vaxijen server 2.0 predicted about 15 proteins as a probable antigen candidate with a threshold of 0.50 for higher sensitivity and accuracy. The scores of the remaining nine proteins were below the threshold value, and thus, they were excluded. The result is shown in **Table 4**.

**Table 4:** The antigenic properties determination using the VaxiJen server.

Serial No	Accession No	VaxiJen Score
**1**	WP_005408386.1	0.5815 (Probable ANTIGEN)
**2**	WP_005409007.1	0.5459 (Probable ANTIGEN)
**3**	WP_005410539.1	0.5306 (Probable ANTIGEN)
**4**	WP_005410716.1	0.4253 (Probable NON-ANTIGEN)
**5**	WP_005411349.1	0.5427 (Probable ANTIGEN)
**6**	WP_005412620.1	0.8651 (Probable ANTIGEN)
**7**	WP_005413200.1	1.1056 (Probable ANTIGEN)
**8**	WP_005413412.1	0.7023 (Probable ANTIGEN)
**9**	WP_005414366.1	0.501 (Probable ANTIGEN)
**10**	WP_012478637.1	0.3267 (Probable NON-ANTIGEN)
**11**	WP_012478648.1	0.5506 (Probable ANTIGEN)
**12**	WP_012478875.1	0.4504 (Probable NON-ANTIGEN)
**13**	WP_012479125.1	0.6294 (Probable ANTIGEN)
**14**	WP_012479796.1	0.4975 (Probable NON-ANTIGEN)
**15**	WP_012479842.1	0.504 (Probable ANTIGEN)
**16**	WP_012479848.1	0.4515 (Probable NON-ANTIGEN)
**17**	WP_012480806.1	0.5217 (Probable ANTIGEN)
**18**	WP_012480920.1	0.595 (Probable ANTIGEN)
**19**	WP_012480949.1	0.4533 (Probable NON-ANTIGEN)
**20**	WP_012481043.1	0.468 (Probable NON-ANTIGEN)
**21**	WP_024956629.1	0.42 (Probable NON-ANTIGEN)
**22**	WP_032966398.1	0.7985 (Probable ANTIGEN)
**23**	WP_044569343.1	0.6115 (Probable ANTIGEN)
**24**	WP_044570756.1	0.4801 (Probable NON-ANTIGEN)

The cutoff was 0.5, which means less than that value is probable non-antigenic in nature.

### Protein-protein interaction

**Fig 2:**
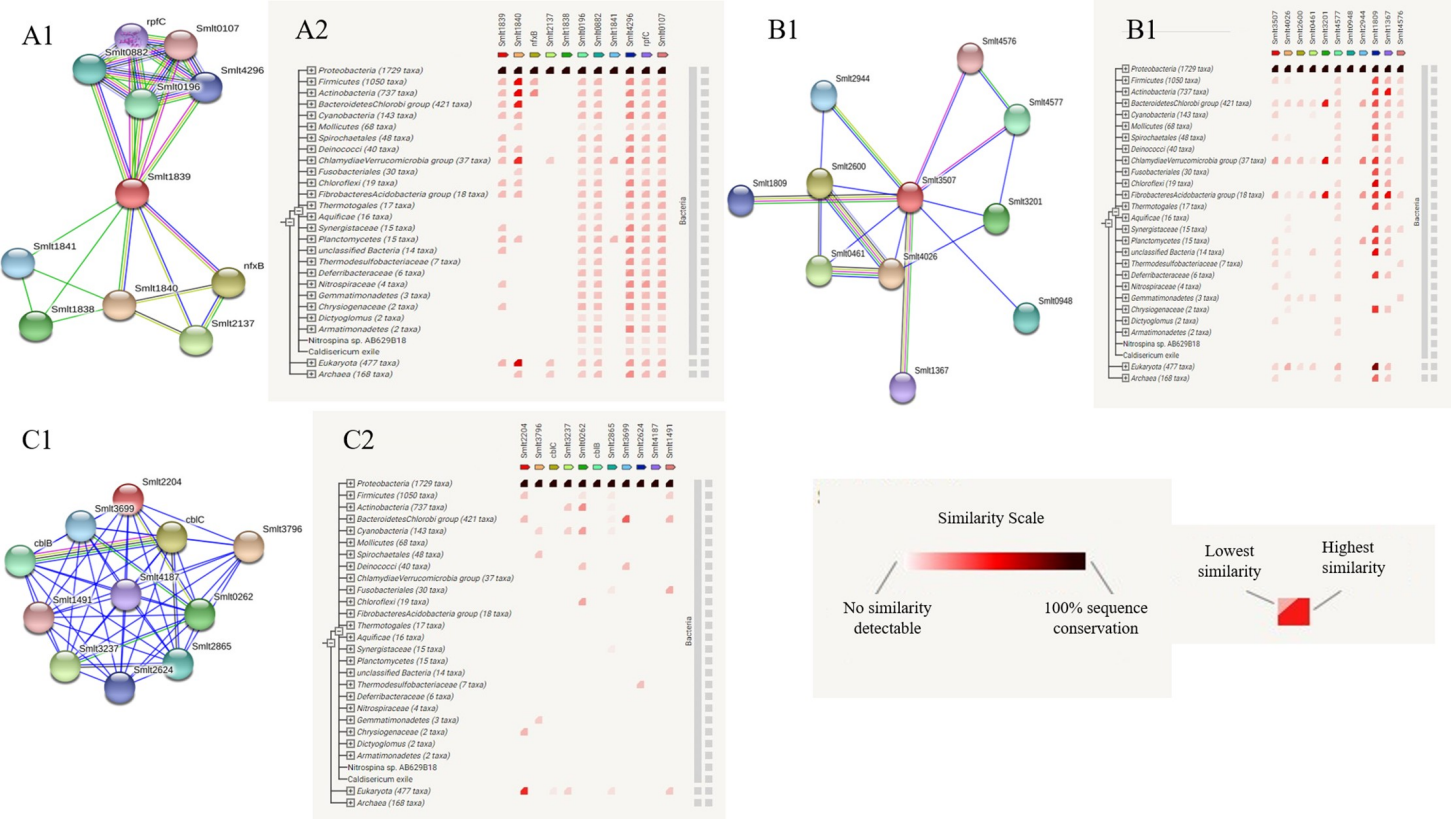
The protein-protein interaction network and gene co-occurrence. 2A1, 2B1 and 2C1 represents the PPI and 2A2, 2B2 and 2C2 represents the gene co-occurrence of WP_012479796.1, WP_012480949.1 and WP_005413200.1 respectively. The color intensity indicates the similarity level.

Interaction between various proteins plays a crucial role in all most all biological processes. One protein mutually interacts with others to perform common cellular functions. For example, the activation of transcription includes multiple transcription factors that work together in gene expression. Moreover, the functions of proteins can be predicted using their PPI information because it is very rare for a protein to interact with different biomolecules. Therefore, PPI databases have become an important resource to study biological networks and pathways [173]. We predicted the PPI and gene co-occurrence for three annotated HCL HPs (highest antigenic protein and two virulent protein), which are thought to be vital players in the pathogenesis of the organism (**Fig 2**). Gene Co-occurrence network is the graphical visualization of a particular gene network that is possibly present, not necessarily conserved, in a variety of biological organisms. Here in the figure, A1, B1, and C1 are the PPI network of the protein WP_012479796.1, WP_012480949.1, and WP_005413200.1, respectively, while A2, B2, and C2 depicted their corresponding gene co-occurrences. The colored nodes of the PPI network represent functionally associated first shell proteins, and each edge shows the type of interactions.

On the other hand, Gene co-occurrence is presented as a phylogenetic tree where the topmost part contains the proteins of the specific network, and the left side contains the organisms. Right-sided color denotes the similarities for a particular gene of interest in a given genome. Higher the color intensity, the higher the sequence similarity or conservancy. For a clade that is collapsed in the tree, the highest and lowest similarities are indicated by two distinct colors.

### Epitope prediction for vaccine target

For the prediction of subunit vaccine candidates, the outer membrane proteins of the bacteria are the target of choice. We have selected only the outer membrane/periplasmic/extracellular proteins predicted by the CELLO prediction tool. We have found 11 such proteins (**S4 Table**). Though each of them can induce the immune response in the host, we selected only the highest antigenic protein (WP_005413200.1 scored 1.1056 in VaxiJen) for this purpose.

#### T cell epitope prediction

NetCTL server identified potential T cell epitopes with preselected criteria using the selected antigenic protein. Seven epitopes that have a combinatorial score of more than 1.5 were selected, and the data is presented in **Table 5**.

**Table 5:** NetCTL T cell epitope prediction with the combinatorial score.

Serial No.	Epitopes	Overall Score (nM)
**1**	RRFDVAQPT	2.3005
**2**	ERAASGAKY	1.8535
**3**	VPSLLAASL	1.8151
**4**	RQYHGCGNF	1.8062
**5**	RATGNEPGW	1.7780
**6**	WTKGSDDGL	1.7301
**7**	RTFAMSSER	1.7202

Using the SMM method, we predicted the MHC-I binding affinity for all of the seven epitopes. A broad range of MHC Class I alleles was screened for interaction with the epitopes. The lower or higher IC50 value measured the affinity. The lower the IC_50_ higher the affinity, and vice versa. We allowed only those MHC-I alleles that interacted with the epitopes with an IC_50_ value of less than 250nM (**Table 6).**

**Table 6:** Promising T cell epitopes with their properties: IC_50_ value, docking score (kcal/mol), combinatorial processing score.

Serial No.	Peptide	Interacting MHC class-I allele	Docking Score i.e Binding affinity (kcal/mol)	IC50 Value <250nM	The combined score of Proteasome score, TAP score, MHC-I score, processing score	Allergenicity
1	RRFDVAQPT	HLA-C*12:03	-8.2	16.72	-0.70	NON-ALLERGEN
		HLA-C*03:03	-8.4	104.86	-1.50	
		HLA-C*14:02	-7.1	113.66	-1.53	
		HLA-B*27:05	-7.1	115.50	-1.54	
		HLA-C*07:02	-8.4	165.26	-1.70	
		HLA-C*07:01	-8.3	194.16	-1.77	
2	ERAASGAKY	HLA-C*03:03	-8.9	32.78	1.02	NON-ALLERGEN
		HLA-C*12:03	-8.2	44.38	0.89	
		HLA-B*15:02	-8.8	49.46	0.85	
3	VPSLLAASL	HLA-C*03:03	-7.1	25.98	0.37	NON-ALLERGEN
		HLA-B*07:02	-7.1	45.28	0.13	
		HLA-C*12:03	-8.1	75.03	-0.09	
		HLA-B*15:02	-9.4	132.20	-0.34	
4	RQYHGCGNF	HLA-B*15:01	-8.7	31.48	1.05	NON-ALLERGEN
		HLA-C*12:03	-9.4	59.32	0.77	
		HLA-C*03:03	-8.8	72.21	0.68	
		HLA-A*32:01	-9.7	121.32	0.46	
		HLA-B*15:02	-9.3	159.30	0.34	
		HLA-C*14:02	-8.1	197.07	1.62	
5	RATGNEPGW	HLA-C*03:03	-10.4	5.96	1.07	NON-ALLERGEN
		HLA-B*58:01	-9.8	12.34	0.76	
		HLA-C*12:03	-10	13.78	0.71	
		HLA-B*57:01	-9	53.07	0.12	
		HLA-B*53:01	-8.6	164.77	-0.37	
6	WTKGSDDGL	HLA-C*12:03	-8	17.43	0.54	ALLERGEN
		HLA-B*15:02	-9.4	25.42	0.38	
		HLA-C*03:03	-8	29.35	0.32	
7	RTFAMSSER	HLA-A*31:01	-8.4	7.20	0.79	NON-ALLERGEN
		HLA-A*68:01	-8.4	13.92	0.51	
		HLA-C*12:03	-6.9	15.28	0.47	
		HLA-C*15:02	-8.8	40.97	0.04	
		HLA-C*03:03	-7.8	48.71	-0.04	
		HLA-A*11:01	-8.6	92.11	-0.31	
		HLA-A*30:01	-8	117.75	-0.42	
		HLA-C*14:02	-7.4	217.58	-0.69	
		HLA-A*03:01	-7	234.86	-0.72	

Allergenicity results of these epitopes are also included.

The IEDB tool predicted MHC-I processing (TAP transport, proteasomal cleavage, and MHC-I combined predictor) with a combined score for individual epitopes from the submitted protein sequence. Peptides are formed due to the cleavage of peptide bonds with the help of the proteasome complex. Then these peptides bind to the MHC Class I molecules and are transported by the TAP proteins to the plasma membrane and presented to the CD4^+^ T cells or helper T lymphocytes. Higher the combinatorial score higher the processing potency (**Table 6**).

The 9 mer peptide RTFAMSSER interacted with the maximum number of alleles among the seven epitopes. The interacted alleles include HLA-A*31:01, HLA-A*68:01, HLA-C*12:03, HLA-C*15:02, HLA-C*03:03, HLA-A*11:01, HLA-A*30:01, HLA-C*14:02 and HLA-A*03:01 (**Table 6**).

#### Allergenicity assessment and population coverage

To avoid cross-reactivity, all the epitopes were subjected to AllerTOP v2.0, and AllerCatPro and six epitopes were predicted as non-allergens by these servers where epitope WTKGSDDGL found to have allergic activity (**Table 6)**. So, we excluded that epitope for further analysis.

**Fig 3:**
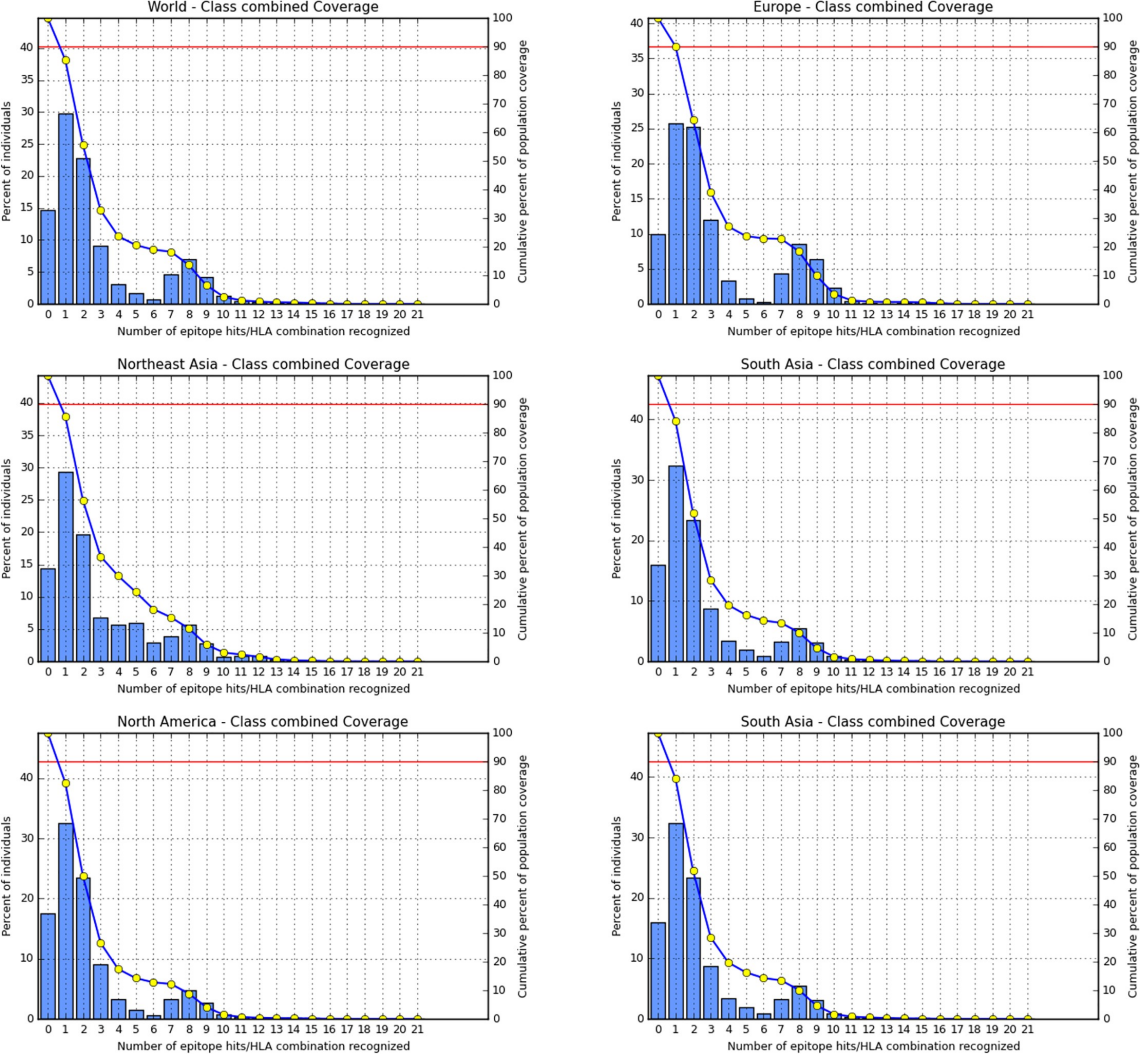
Population coverage data based on MHC class I restriction data. Here the line (-o-) shows the cumulative percentage of population coverage for the epitopes, and the bars represent individual population coverage of the epitopes.

Population coverage is a crucial parameter in vaccine development. Hence, the cumulative population coverage percentage was measured using the IEDB population coverage tool for all the non-allergenic epitopes. We found the maximum coverage in Europe, which was 90.03%, followed by Northeast Asia 85.65%, and South Asia 84.06%. Besides, we also measured the population coverage for North America (82.53%) and Southeast Asia (80.64%). The cumulative World population coverage was 85.30%. The results are summarized in **Table 7** and **Fig 3**

**Table 7:** Population coverage results of the epitopes using IEDB resource.

Country/Region	Coverage	Average hit	PC90
**World**	85.30%	2.81	0.68
**Europe**	90.03%	3.25	1
**Northeast Asia**	85.65%	2.92	0.7
**South Asia**	84.06%	2.46	0.63
**North America**	82.53%	2.32	0.57
**Southeast Asia**	80.64%	2.29	0.52

#### Molecular docking analysis

**Fig 4:**
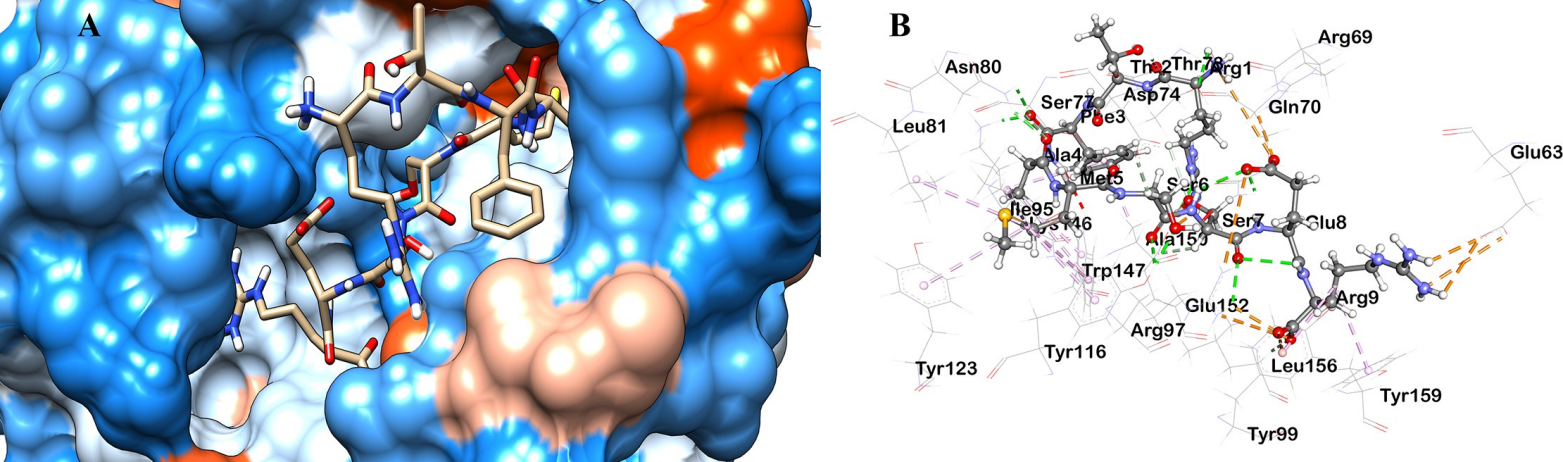
Predicted docking mode analysis of the HLA-C*03:03 and the epitope RTFAMSSER where the epitope binds to the binding cleft of the HLA. Here, (A) Pose in solid surface mode (B) Binding orientation map.

Molecular docking is the most common method used in reverse vaccinology to analyze the interaction pattern between epitopes and MHC molecules. We performed molecular docking in general for all the epitopes and the respective alleles (**Table 6**). The ranges of docking score, i.e., binding affinity was between -6.9 to -10.4 kcal/mol, respectively. The IC_50_ values were taken for the study were <250 nM, which is an indication of strong binding affinity between alleles and their respective epitopes. The higher the IC50, the lower the affinity [105]. Along with that, the combined scores of proteasome score, TAP score, MHC-I score, processing score are a quantity-based prediction that is proportional to the total amount of peptides presented by the MHC molecules on the surface of the cells. Higher the value, the higher the amount of presented peptides [105]. The IC50 value, combined scores, and docking scores cumulatively showed a strong interaction pattern between the epitopes and the HLA with an average binding affinity of -8.4 kcal/mol. Though all the non-allergen epitopes can individually induce an immune response, we took only the RTFAMSSER epitope for post docking interaction analysis because it interacted with the maximum number of alleles as compared to others. To check the interaction modes between the predicted T-cell epitope and the HLA-C*03:03, molecular docking was performed using Autodock Vina. The result comes with a binding affinity of -7.8 kcal/mol. In addition, our study about the HLA-C*03:03 suggests that the binding cleft of the MHC molecule is located near the α1 helix region between residues 70–77 and α2 helix region between residues 144–152 [174]. Post docking interaction was analyzed **(Fig 4),** and the nonbonding interaction data are tabulated in **Table 8.**

**Table 8:** Nonbonding interactions with their distances (Å) between epitope (RTFAMSSER) and HLA (HLA-C*03:03).

Hydrophobic		Hydrophobic		Electrostatic	
Alkyl	Pi-Alkyl	Conventional	Salt Bridge	Attractive Charge	Pi-Anion
LYS^146^ (4.225) LEU^81^ (5.296) ILE^95^ (4.974)	LYS^146^ (5.329) ALA^150^ (3.880) TRP^147^ (4.293) TRP^147^ (5.492) TYR^116^ (4.653) TYR^123^ (5.237) TRP^147^ (5.366) TYR^159^ (4.428) TYR^99^ (4.617)	GLU^152^ (2.757) THR^73^ (2.893) ASN^80^ (2.390) LYS^146^ (2.157) ASN^80^ (2.118) TYR^116^ (2.695) TYR^116^ (2.015) ARG^97^ (2.389) ARG^97^ (2.471) GLN^70^ (2.352)	GLU^152^ (2.131) ARG^69^ (2.621) ARG^97^ (2.451) GLU^63^ (2.882) GLU^63^ (2.581)	GLU^152^ (2.131) ARG^69^ (2.621) ARG^97^ (5.575) GLU^63^ (2.882) GLU^63^ (2.581) ARG^97^ (2.451) ARG^97^ (4.332) GLU^63^ (4.252)	TYR^99^ (3.490)

Post docking analysis of the docked complex shows that our potent epitope formed 12 hydrophobic, nine electrostatic, and 15 hydrogen bonds with the MHC molecule. Half of the hydrophobic interaction was formed within the binding cleft, which is an indication of the stable binding pattern as combined hydrophobic interaction plays a vital role in protein stability. In hydrophobic interaction, epitope interacted with the MHC molecule only in α2 binding cleft where interestingly hydrogen bond was formed in both α1 and α2 binding cleft. Interestingly, Lys146 showed both alkyl and pi-alkyl type hydrophobic interaction along with conventional hydrogen bond, whereas Glu152 exhibited salt bridge and conventional hydrogen bond along with attractive charge type electrostatic interaction. Experimental evidence shows that the dimorphic amino acid Asn80 generally interacts with both NK cell receptors and the foreign antigens (epitopes) [174]. Interestingly, this docking result shows two conventional hydrogen bonds between Asn80 and the epitope.

#### B cell epitope prediction

**Fig 5:**
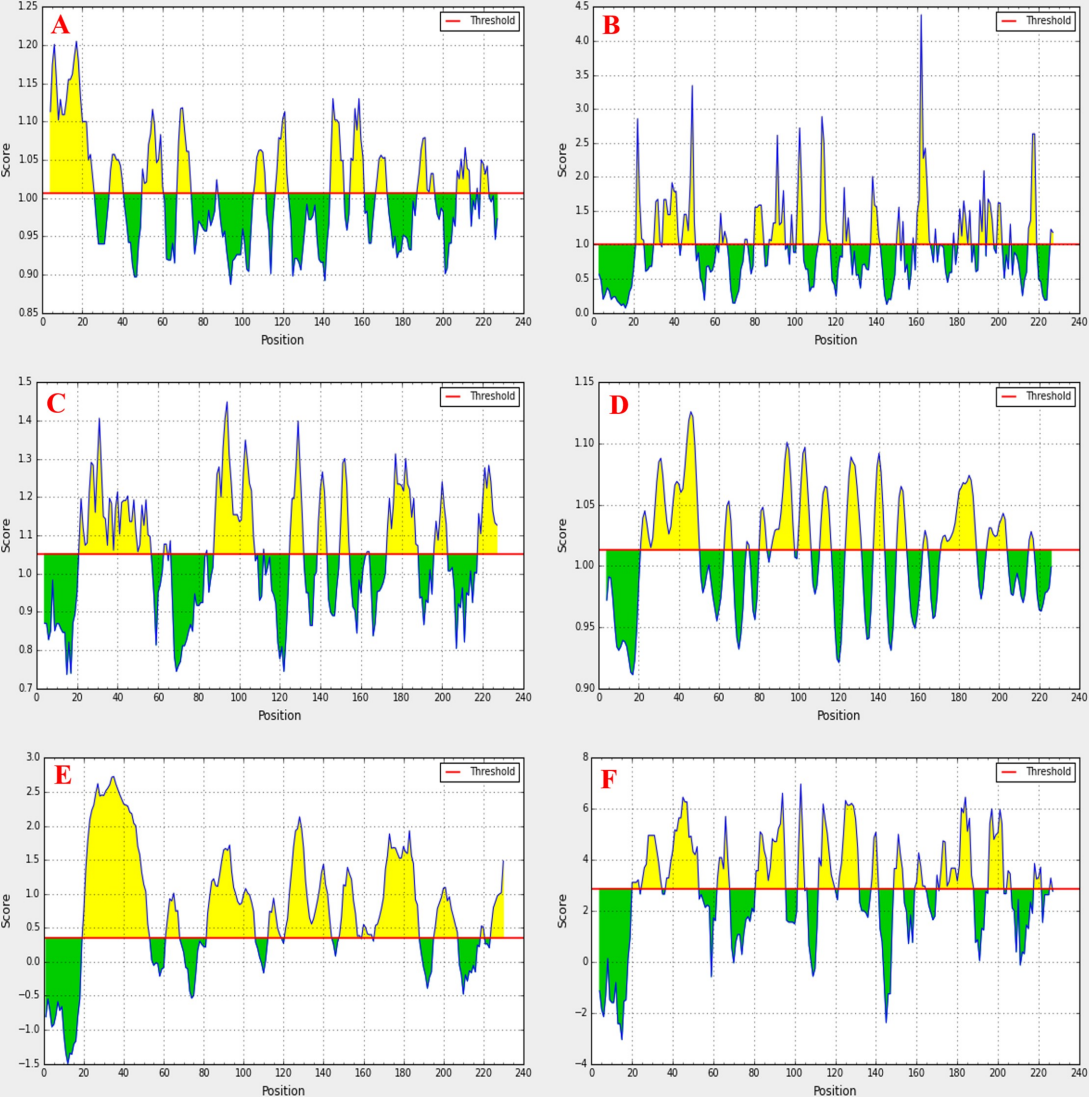
Prediction of B cell epitope properties for the conserved antigenic region. Region 34–42 (PAAPQPSAS) possessed the maximum antigenic criteria as a potential B cell epitope. (A) Kolaskar and Tongaonkar antigenicity prediction, (B) Emini surface accessibility prediction. (C) Chou and Fasman beta-turn prediction, (D) Karplus and Schulz flexibility prediction, (E) Bepipred linear epitope prediction, (F) Parker hydrophilicity prediction. The regions with antigenic nature are shown in yellow color.

*Linear B cell epitope prediction*. Several authentic tools were recruited to identify potential linear B-cell epitopes (**Fig 5**). Kolaskar and Tongaonkar antigenicity prediction tool assessed the conserve regions considering the Physico-chemical properties of the protein. The threshold value was set at 1.00, which determines the possibility of a conserved region being a potential antigen scoring more than that. The minimum and maximum antigenic propensity values were 0.920 and 1.240, where the average was 1.058. We were able to identify such regions that can induce a humoral immune response presented in **Fig 5A and Table 9**.

**Table 9:** Predicted epitope from Kolaskar and Tongaonkar antigenicity prediction tool.

No.	Start	End	Peptide Sequence	Length
**1**	4	25	VPSLLAASLGLVLAACQPAQPP	22
**2**	34	40	PAAPQPS	7
**3**	50	60	TYQCGDLSVRA	11
**4**	68	74	ATVVIGE	7
**5**	106	111	GLLSLK	6
**6**	117	122	ECHAVE	6
**7**	144	150	WLAVVDG	7
**8**	154	160	GLQVEVD	7
**9**	167	172	DVAQPT	6
**10**	187	195	DVKLSFQRT	9
**11**	207	213	DAKVNLT	7

We were ascertained of the region 35–42 amino acid residues as surface accessible by the Emini Surface Accessibility prediction tool that can act as B cell epitope (**Fig 5B and Table 10**).

**Table 10:** Results from Emini surface accessibility prediction.

Serial No	Start	End	Peptide	Length
**1**	35	42	AAPQPSAS	8
**2**	44	50	EGGSETT	7
**3**	87	94	GAKYGDGK	8
**4**	112	117	GEADRE	6
**5**	160	166	DYGERRF	7
**6**	181	186	KASDGT	6

Moreover, it is well known about the surface accessibility or hydrophilicity of the beta-turn regions of a protein, which was predicted by Chou and Fasman Beta-turn prediction tool. The predicted beta-turn regions were 20–57, 87–107, and 171–188 (**Fig 5C**). The antigenicity of the peptide is strongly correlated with its flexibility [100]. Karplus and Schulz flexibility prediction tool identified 21–51 as the most flexible regions (**Fig 5D**). In the end, Bepipred linear epitope prediction tool suggested the probable linear B-cell epitopes (**Fig 5E and Table 11**).

**Table 11:** Bepipred linear epitope prediction result.

Serial No.	Start	End	Peptide Sequence	Length
**1**	20	52	QPAQPPAAGGNDAPPAAPQPSASTEGGSETTYQ	33
**2**	61	67	TFNGEDA	7
**3**	82	105	ERAASGAKYGDGKGNSFWTKGSDD	24
**4**	113	118	EADREC	6
**5**	121	144	VEATEGDGSAGNAAFRATGNEPGW	24
**6**	148	158	VDGDTPGLQVE	11
**7**	160	164	DYGER	5
**8**	166	187	FDVAQPTAGADGWSGKASDGTD	22
**9**	196	207	TCQDDMSGEAFD	12
**10**	219	220	YH	2

Parker Hydrophilicity prediction tool was recruited for further confirmation about the hydrophilic nature of our predicted B cell epitopes (**Fig 5F**). Analysis of the data from B cell epitope prediction tools revealed the most potent B cell-mediated immunity inducing conserved epitope ’PAAPQPSAS’ in the region of 34–42.

### Conformational B cell epitope prediction

Most of the epitopes for B cells are discontinuous or conformational rather than linear [175]. To predict the discontinuous B cell epitopes, the 3D structure of the protein was generated and validated, and submitted to the Ellipro server. This server identified eight different epitopes for the protein WP_005413200.1 (**Table 12**).

**Table 12:** Amino acid residues of the conformational B cell epitopes.

Serial No.	Conformational B cell epitope residues	Number of residues	Score
**1**	MET^1^, ARG^2^, VAL^3^, VAL^4^, VAL^210^, ASN^211^, LEU^212^, THR^213^, ILE^214^, GLY^215^, THR^216^, ARG^217^	12	0.796
**2**	ALA^173^, SER^179^, GLY^180^, LYS^181^, ALA^182^, SER^183^, ASP^184^, GLY^185^, THR^186^, ASP^187^, VA^188^, LYS^189^, LEU^190^, SER^191^, PHE^192^, GLN^193^, THR^195^, THR^196^, CYS^197^, GLN^198^, ASP^199^, ASP^200^, MET^201^, SER^202^, GLN^203^, GLU^204^, ALA^205^, PHE^206^, ASP^207^, ALA^208^, LYS^209^, ALA^227^, LYS^228^, GLN^229^, PRO^230^	35	0.714
**3**	ALA^22^, GLN^23^, PRO^24^, PRO^25^, ALA^26^, ALA^27^, GLY^28^, GLY^29^, ASN^30^, ASP^31^, ALA^32^, PRO^33^, PRO^34^, ALA^35^, ALA^36^, PRO^37^, PRO^39^, SER^40^, ALA^41^	19	0.676
**4**	THR^50^, TYR^51^, GLN^52^, CYS^53^, GLY^54^, ASP^55^, LEU^56^, SER^57^, VAL^58^, ARG^59^, VAL^71^, ILE^72^, GLY^73^, GLU^74^, ARG^75^, THR^76^, PHE^77^, ASP^104^, SER^109^, LEU^110^, LYS^111^, GLY^112^, GLU^113^, ALA^114^, ASP^115^, ARG^116^, GLU^117^, CYS^118^, HIS^119^, ALA^120^, VAL^121^, GLU^122^, ALA^123^, THR^124^, GLU^125^	35	0.646
**5**	GLY^93^, LYS^94^, GLY^95^, ASN^96^	4	0.642
**6**	GLN^218^, TYR^219^, HIS^220^, GLY^221^	4	0.626
**7**	GLY^126^, ASP^127^, GLY^128^, SER^129^, GLY^154^, LEU^155^, GLN^156^, VAL^157^, GLU^158^, VAL^159^, ASP^160^, TYR^161^, GLY^162^, GLU^163^, ARG^164^, PHE^166^, ASP^167^, VAL^168^, ALA^169^, GLN^170^, PRO^171^, GLY^174^	22	0.582
**8**	GLY^223^, ASN^224^, PHE^225^	3	0.503

**Fig 6:**
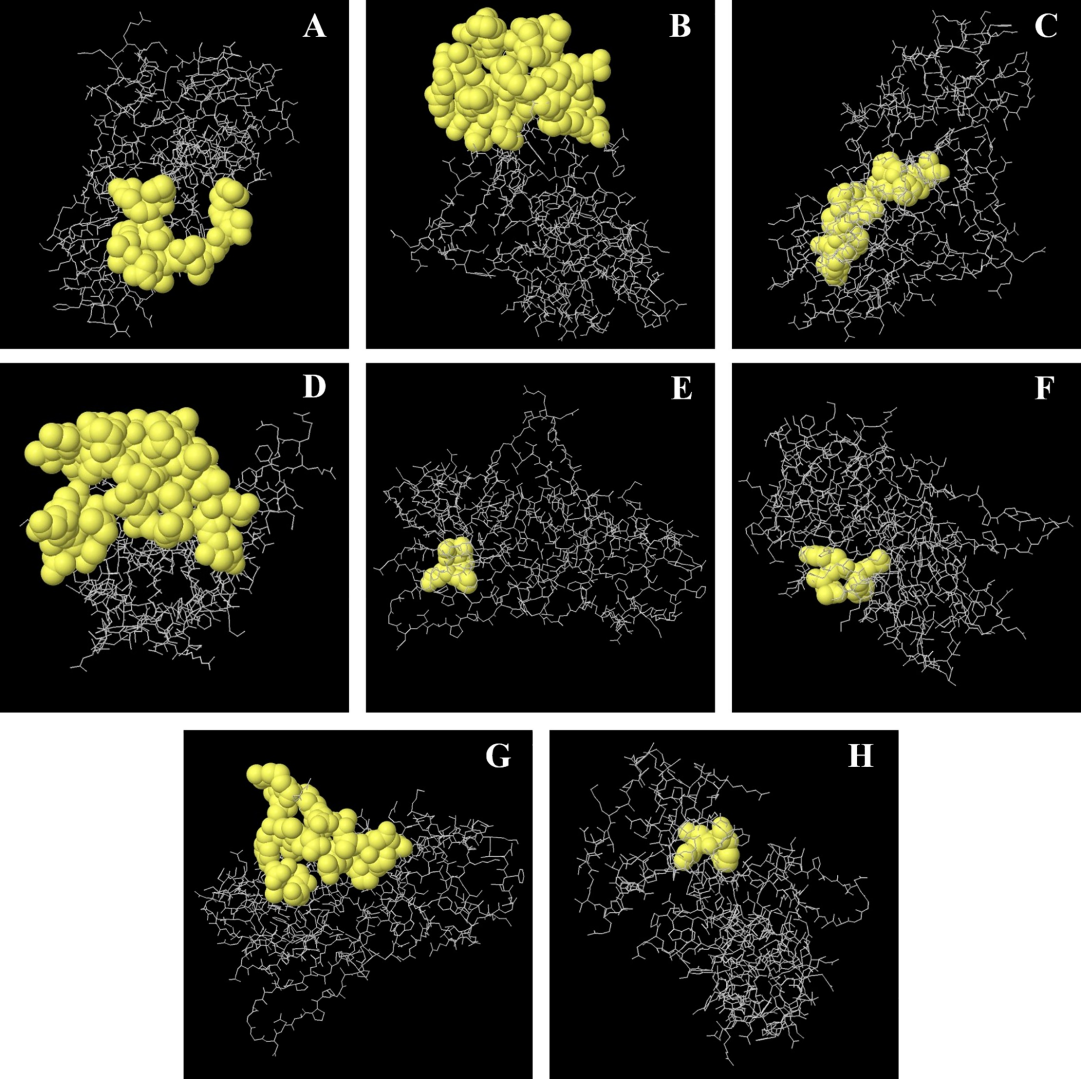
Conformational or discontinuous B cell epitopes of WP_005413200.1 predicted from the PDB structure (homology). Here, A-H, the yellow balls represent the residues of the corresponding epitopes, and sticks in white color are the structure of the core residues.

The 3D structures of these epitopes were visualized using Jmol (integrated service of the server), which demonstrates their particular positions in the protein. The epitope residues were predicted using the full-length protein, where they were scattered throughout the surface. The scores of the predicted epitopes reside between 0.503 to 0.796, where the cutoff score was previously selected 0.50 by default. The detailed view of these conformational epitopes is illustrated in **Fig 6**.

There are still some limitations in epitopes prediction using different bioinformatics tools, and therefore, improvements are required in the prediction methods. Improving the incorrectly delineated epitope databases can result in higher accuracy prediction [176]. It is more suitable to include multiple tools for more accurate and consistent outcomes as the results obtained from different tools and methods may differ [177].

## Conclusions

At first, we resolved all the 789 HPs from *S*. *maltophilia K279a* and predicted the functions with precision and confidence for 24 proteins. Next, the characterization was carried out, followed by the functional validation with different approaches, including structure-based methods. The physical and chemical parameters and the subcellular localization information helped to distinguish the HPs from the others. The PPI also gave an idea about their corresponding metabolic pathways. Besides, we were able to detect two virulence-associated proteins vital for the pathogenesis and survival of this organism. Among the HPs, we predicted the T cell and B cell epitopes for the highest antigenic protein, which is located in the periplasmic membrane of the pathogen. Pieces of evidence of our study suggest the potency of these epitopes as good targets against the bacteria. Nevertheless, clinical experiments are needed to ensure the efficacy of these candidates as vaccines.

## Supporting information

S1 Table The conserved domain analysis result of all the 789 HPs of *S*. *maltophilia*. (XLSX)

S2 Table The predicted physico-chemical properties of the proteins using Expasy’s ProtParam. (XLSX)

S3 Table Structural and functional association prediction by PS2 v-2 and STRING. (XLSX)

S4 Table Predicted result for subcellular localization using various servers. (XLSX)
